# Fundamental Understanding and Quantification of Capacity Losses Involving the Negative Electrode in Sodium‐Ion Batteries

**DOI:** 10.1002/advs.202306771

**Published:** 2023-12-07

**Authors:** Le Anh Ma, Alexander Buckel, Andreas Hofmann, Leif Nyholm, Reza Younesi

**Affiliations:** ^1^ Department of Chemistry‐Ångström Laboratory Uppsala University Uppsala SE‐75121 Sweden; ^2^ Karlsruher Institut für Technologie Institut für Angewandte Materialien (IAM) Herrmann‐von‐Helmholtz Platz 1 76344 Eggenstein‐Leopoldshafen Germany

**Keywords:** ageing, electrolytes, sodium‐ion batteries, solid electrolyte interphase

## Abstract

Knowledge about capacity losses related to the solid electrolyte interphase (SEI) in sodium‐ion batteries (SIBs) is still limited. One major challenge in SIBs is that the solubility of SEI species in liquid electrolytes is comparatively higher than the corresponding species formed in Li‐ion batteries. This study sheds new light on the associated capacity losses due to initial SEI formation, SEI dissolution and subsequent SEI reformation, charge leakage via SEI and subsequent SEI growth, and diffusion‐controlled sodium trapping in electrode particles. By using a variety of electrochemical cycling protocols, synchrotron‐based X‐ray photoelectron spectroscopy (XPS), gas chromatography coupled with mass spectrometry (GC‐MS), and proton nuclear magnetic resonance (^1^H‐NMR) spectroscopy, capacity losses due to changes in the SEI layer during different open circuit pause times are investigated in nine different electrolyte solutions. It is shown that the amount of capacity lost depends on the interplay between the electrolyte chemistry and the thickness and stability of the SEI layer. The highest capacity loss is measured in NaPF_6_ in ethylene carboante mixed with diethylene carbonate electrolyte (i.e., 5 µAh h^−1/2^_pause_ or 2.78 mAh g·h^−1/2^_pause_) while the lowest value is found in NaTFSI in ethylene carbonate mixed with dimethoxyethance electrolyte (i.e., 1.3 µAh h^−1/2^_pause_ or 0.72 mAh g·h^−1/2^_pause_).

## Graphical abstract

A detailed quantification of different aging mechanisms related to electrolyte solution and negative electrode is presented in this study. The dissolution of solid electrolyte interphase (SEI), diffusion‐controlled ion trapping, continuous SEI dissolution and reformation, and chemical desodiation and SEI growth in sodium‐ion batteries are discussed here using a variety of analytical tools.

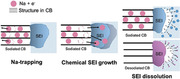

## 1 Introduction

With increasing consciousness about global warming and climate change, the demand for renewable energy technologies followed by sustainable energy storage options is increasing. In this respect, rechargeable batteries based on alkali metal ions such as Li^+^ and Na^+^ have gained much attention for use in different applications.^[^ ^1^, ^2^ ^]^ It is, however, still important to ensure that the performances of these batteries are improved especially regarding their lifetime. The latter is particularly important in applications such as stationary energy storage where long battery lifetimes are required. Therefore, the aging of electrodes and electrolytes as well as the influence of electrode‐electrolyte interfacial reactions need to be more thoroughly comprehended.^[^ ^3^ ^]^ For alkali‐ion batteries, most non‐aqueous electrolytes are unstable at the low electrode potentials of the negative electrode, which is why a passivating layer, known as the solid electrolyte interphase (SEI) layer generally is formed. Ideally, the SEI should be formed during the first cycles under minimum charge consumption to circumvent large irreversible capacity losses. The SEI layer should be ionically conducting to facilitate the migration of alkali‐ions, electronically insulating and impermeable to solvent molecules to avoid continuous solvent reduction. In addition, the SEI should be chemically inert and insoluble in the electrolyte as gradual SEI dissolution would result in a continuous SEI formation, yielding further capacity losses.^[^ ^4^, ^5^, ^6^, ^7^, ^8^ ^]^

Aging processes involving carbon‐based electrodes can be due to a variety of phenomena, such as i) SEI formation and growth resulting in initial capacity losses, ii) gradual cracking of the electrode resulting in a loss of electric contact and an increased cell impedance, iii) diffusion‐controlled Li‐/Na‐trapping, iv) SEI dissolution requiring continuous SEI reformation, and v) chemical deinsertion leading to growth of the SEI thickness.^[^ ^9^, ^10^, ^11^, ^12^, ^13^, ^14^ ^]^ It is often stated that the SEI layers formed in Na‐based electrolyte systems are more soluble than their Li counterparts.^[^ ^7^, ^8^, ^15^ ^]^ It is therefore crucial to improve the understanding of the factors controlling the stabilities of the SEI layers formed in sodium‐ion batteries (SIBs) in order to improve their long‐term performances.

In our previous work, Pt foil electrodes were used as model electrodes to study SEI dissolution by including long pauses (i.e., 50 h) at a high potential (2 V vs Na^+^/Na) after galvanostatic cycling.^[^ ^8^ ^]^ Since at high potentials like 2 V, there is no possibility to reform the SEI layer, we here carry out experiments to understand the formation, dissolution, and consecutive reformation of the SEI layer at lower potentials. At sufficiently low potentials a dissolution of the SEI layer should result in a loss of capacity as the electrode should be able to reduce the electrolyte and maintain the SEI layer intact. Larger capacity losses would then be expected during open circuit pauses when the electrode is sodiated (i.e., reduced) rather than desodiated (oxidized) prior to the pause. To mimic SEI aging on carbonaceous electrodes, high surface area carbon black (CB) electrodes are mainly used.^[^ ^16^ ^]^ However, low surface area hard carbon electrodes were also tested to demonstrate that the same behavior is obtained. The capacity associated with the SEI formation is often overshadowed by the capacity due to the sodiation/desodiation processes in the bulk. The first reason why CB is a particularly suitable electrode material to study the aging mechanism related to SEI is that it has a lower sodiation/desodiation capacity (in this study ≈50–80 mAh g^−1^) than conventional carbon electrodes, composed of hard carbon (HC, 200–300 mAh g^−1^)^[^ ^17^, ^18^ ^]^ or graphite (> 200 mAh g^−1^).^[^ ^19^ ^]^ Secondly, hard carbon and graphite often have relatively low specific surface areas (i.e., 5‐+25 m^2^ g^−1^)^[^ ^18^, ^19^ ^]^ compared to CB, which has a surface area of 62 m^2^ g^−1^. The lower sodiation/desodiation capacity and the larger SEI capacity (due to the larger surface area) for CB thus result in a higher SEI‐to‐sodiation/desodiation capacity ratio.^[^ ^16^ ^]^ In the present case, the sodiation and desodiation capacities result from Na^+^ intercalation/deintercalation into CB, double‐layer charging, and the SEI formation and reformation charges. It will, however, be assumed that the reversible capacity stems from Na^+^ intercalation and deintercalation as the contributions from double‐layer charging typically are small for battery materials.

In half‐cells containing Na‐metal counter electrodes, the CB working electrode is capacity limiting which is why changes in the capacity of the CB electrode can be readily monitored. A difference between the CB reduction and oxidation capacities should be seen in the presence of SEI formation, and the Coulombic efficiency (CE) should hence be lower than 100%. It should, however, be noted that the capacity of the CB electrode should not change as a result of SEI formation as the Na counter electrode will compensate for the SEI charge. In full cells, SEI formation will, on the other hand, result in a consumption of the capacity of the cell via a loss of the capacity of the cathode, assuming that the latter is capacity‐limiting. In other words, while the CE would be lower than 100% in the presence of SEI formation in the half‐cells employed here, the capacity of the CB electrode should remain the same when using a Na‐metal counter electrode. If the charge‐storing capacity of the CB decreases, this would consequently indicate the presence of another phenomenon directly affecting the capacity of the CB electrode. In this case, the CE value would thus be lower than 100% due to both this phenomenon and SEI formation. However, as the term “capacity loss” can refer to either a decrease in the inherent charge‐storing capacity of the CB electrode or a difference between the sodiation (i.e., reduction) and desodiation (i.e., oxidation) capacities, it is important to define what is meant by this term in each study. In this work, the term “capacity loss” refers to the difference between the sodiation and desodiation capacities.

Three different electrolyte salts dissolved in three different ethylene carbonate (EC) based mixtures are used to systematically investigate the stabilities of the SEI layers. Galvanostatic cycling including extended pauses under open circuit voltage (OCV) conditions after the sodiation (i.e., reduction) or desodiation (i.e., oxidation) of the CB electrodes used in combination with gas chromatography coupled with mass spectrometry (GC‐MS), and proton nuclear magnetic resonance spectroscopy (^1^H‐NMR), and synchrotron‐based hard X‐ray photoelectron spectroscopy (XPS) to demonstrate the interplay between the capacity losses, SEI thicknesses, and SEI compositions.

Note that the main aim of this study is to highlight the importance of identifying and quantifying a variety of aging mechanisms related to SEI layer formation on negative electrode materials. The methodology presented can also be used to investigate, not only the nine electrolyte systems studied here but any other electrolyte system.

## 2 Results and Discussion

### 2.1 Galvanostatic Cycling Protocols

**Figure 1:**
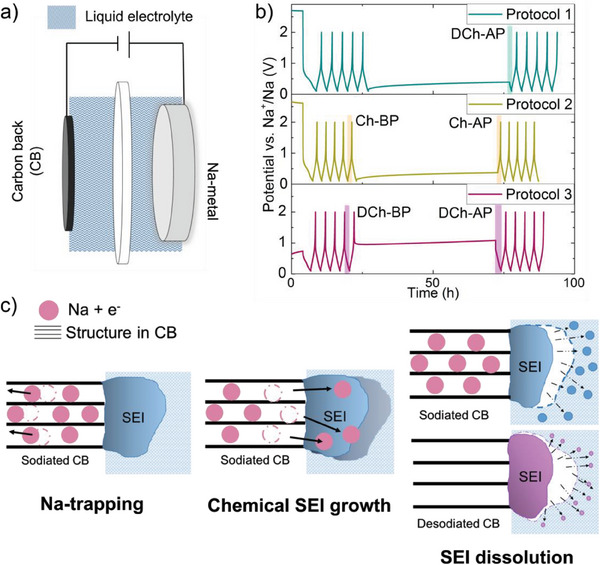
Cell and cycling protocols used to differentiate between capacity losses due to different aging mechanisms. a) Cell setup displaying the CB working electrode and the Na metal counter electrode in a liquid electrolyte with Na‐conductive β‐alumina as the separator. b) Plots of the cell voltage versus time curves, obtained with the three cycling protocols. In all protocols, the cells were cycled five times between 0.1 and 2.0 V with a current density of 63.7 µA cm^−2^ and then stopped at a specific potential and relaxed for 50 h under open circuit conditions. Protocol 1: the cells were stopped at 0.1 V, subjected to the 50‐h open circuit pause, and finally reduced (discharged) to 0.1 V. The capacity loss was determined using the reduction (discharge) capacity after the pause (R‐AP). Protocol 2: the cells were stopped at 0.1 V, subjected to the 50‐h open circuit pause, and finally oxidized (charged) to 2.0 V. The capacity loss was determined using the difference between the oxidation (charge) capacity before (O‐BP) and after the pause (O‐AP). Protocol 3: the cells were stopped at 2.0 V (where a fast relaxation to ≈1 V was observed), subjected to the 50‐h open circuit pause, and finally discharged to 0.1 V. The capacity loss due to a “one‐time” SEI dissolution was calculated using the difference between the reduction capacity before (R‐BP) and after the pause (R‐AP). All the final states after the extended pause are highlighted with color in Figure 2b. c) Schematic illustration of the different aging mechanisms discussed in this work.

**Figure** **1** shows a schematic of the cell setup and the galvanostatic cycling protocols used in this study. CB electrodes were cycled against Na‐metal electrodes in half‐cells containing non‐aqueous electrolytes and Na‐conductive β‐alumina separators to prevent crosstalk between the Na and CB electrodes (see Figure 1a).^[^ ^20^ ^]^ As a side note, the cell setup was evaluated compared to using a conventional separator such as Solupor and hard carbon electrode material; the results and detailed discussion are presented in Figure S1, Supporting Information. In brief, the use of a conventional separator such as Solupor allows cross‐talk between the electrodes and thus diffusion of electrolyte decomposition species from the Na electrode to the CB electrode and to the electrolyte solution. Therefore, the use of a β‐alumina separator limits the adverse effect of the Na metal counter electrode while not influencing the cycling profiles (see Figure S1, Supporting Information).

Three cycling protocols were used as schematically presented in Figure 1b; each cell first was cycled with a constant current of 50 µA (63.7 µA cm^−2^) five times between 0.1 and 2.0 V versus Na^+^/Na (all potentials are hereafter reported vs Na^+^/Na), paused at either 0.1 or 2.0 V subjected to a 50‐h open circuit pause (see Figure 1b). While longer open circuit pauses could have been used, the abovementioned pause was found to be sufficiently long to yield significant capacity losses. The purpose of this open circuit pause testing at different potentials was to determine the capacity losses originated from a variety of aging mechanisms that are independent of net charge transfer (i.e., occur during storage with no applied potential or current). The lower cut‐off potential was set to allow electrolyte reduction and Na^+^ insertion into the CB, whereas the upper cut‐off voltage was set to assess the capacity losses at a potential where no SEI reformation on the CB electrode was possible.

In the first protocol (protocol 1, Figure 1b), the cell was first paused for 50 h at 0.1 V and then the CB electrode was reduced (i.e., sodiated/discharged) again to 0.1 V after the pause (see Figure 1b). Here the reduction (sodiation/discharge) capacity obtained after the open circuit pause (R‐AP) is due to the deinsertion of Na^+^ since some of the inserted sodium was consumed during the relaxation time. Several different mechanisms can yield such aging; i) reaction of the reduced carbon with the electrolyte to reform the SEI continuously to counteract the SEI dissolution, ii) electron leakage via the SEI leading to further reduction of the electrolyte solution resulting in growth of SEI thickness, and iii) diffusion of inserted Na into the core of the carbon particles.

**Figure 2:**
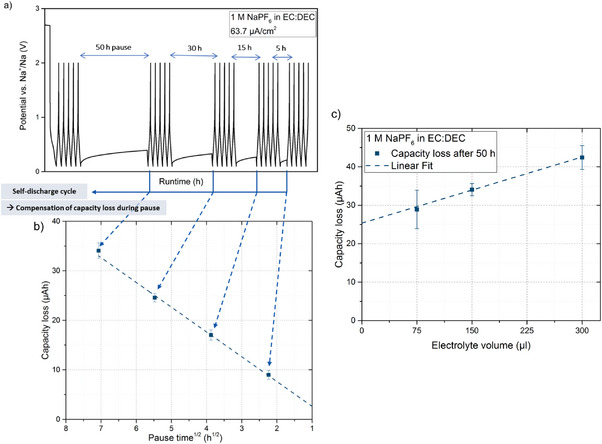
Galvanostatic cycling according to procedure 1 including differently open circuit pauses using 150 µl of 1 M NaPF_6_ dissolved in EC:DEC. a) The cell voltage profile, b) The capacity loss after an open circuit pause as a function of the square root of the pause time, indicating a capacity loss due to a diffusion‐controlled process, and c) The capacity obtained after a 50 h long pause as a function of the electrolyte volume. The error bars represent the standard deviations obtained based on the results from three replicate cells.

Using protocol 1, we then measured capacity losses during storage (pause time) for a cell with 1 M NaPF_6_ in EC:DEC electrolyte cycled with different open circuit pause times, that is, 50, 30, 15, and 5 h (see **Figure** **2**). The results reveal that the measured capacity loss has a linear dependence on the square root of the pause time as shown in Figure 2b, which implies that the capacity loss was coupled to a diffusion‐controlled process. Analogous plots have previously been reported for lithium‐based batteries.^[^ ^13^ ^]^ As is shown in Figure 2c, a linear plot was also obtained when plotting the capacity loss versus the electrolyte volume for the measured data points. The behavior is caused by the dissolution of the SEI components as more SEI needs to undergo dissolution in order to saturate a larger volume of electrolyte. SEI dissolution therefore had a clear impact on the total capacity loss observed after the 50‐h open circuit pause.

Another important message from Figure 2c is the fact that the intercept of the extrapolated line with the *y*‐axis is not zero since it shows a capacity loss of almost 25 µAh for an electrolyte volume of zero µl. This means that the measured capacity loss cannot only be explained by the SEI dissolution. Such capacity loss could therefore be assigned to other aging mechanisms such as i) chemical formation and growth of SEI involving no external current or potential, and ii) diffusion‐controlled redistribution of inserted sodium in CB electrode which can also lead to trapping of sodium into the core of active material particles. The driving force for the former is the chemical potential difference between sodium in the CB electrode and oxidized sodium in the SEI, while the driving force for the latter is the concentration gradient of sodium at the surface and in the core of the CB particles.^[^ ^13^, ^14^ ^]^ Figure S2, Supporting Information shows that such trapped sodium ions can be mostly desodiated if the current density is significantly lowered (i.e., longer time is given for desodiation using lower current density). The aforementioned charge losses lead to an increase in the potential of the sodiated CB electrode during the pause time as can be seen in Figure S3a, Supporting Information.

In the second protocol (protocol 2, Figure 1b), the CB electrode was paused at a low potential of 0.1 V and then oxidized (i.e., desodiated/charged). The difference between the oxidation (charge) capacity before the pause (O‐BP) and after the pause (O‐AP) reflects the capacity lost during the relaxation. The capacity losses seen with protocols 1 and 2 should therefore be due to the same aging process involving continuous SEI dissolution and reformation, electron leakage and the subsequent SEI growth, and diffusion‐controlled trapping of Na in the carbon particles. In the third protocol (protocol 3, Figure 1b), where the cell was paused at a high potential, that is, 2 V, prior to discharge, the measured reduction capacity after the pause (R‐AP) should be ascribed to the re‐insertion of Na into the CB structure and the SEI formation needed to make up for the SEI dissolution during the open‐circuit pause. In this case, the CB electrode was thus oxidized prior to the open circuit pause and subsequently reduced. During the open circuit period, there should hence only be a “one‐time” SEI dissolution and no reformation since the oxidized CB electrode cannot reduce the electrolyte. The difference between the reduction capacities before the pause (R‐BP) and after the pause (R‐AP) should consequently mainly represent the capacity loss due to the “one‐time” SEI dissolution process (see Figure 1c). The capacity losses due to Na‐trapping and chemical desodiation should therefore be negligible in this case.

**Table** **1** presents a summary of the capacity losses seen for two different electrolytes, that is, 1 M NaPF_6_ in EC:DEC and 1 M NaTFSI in EC:DEC when using the three aforementioned cycling protocols. It is clear that while the results from the first and second cycling protocols were similar, the losses seen for the third protocol were significantly smaller than those for protocols 1 and 2, for both electrolytes. There were also no significant differences between the results for the two electrolytes for any of the protocols. This is in good agreement with the experimental data as the magnitude of the uncertainties obtained from three replicate cells indicate that the differences between the values were not significant.

**Table 1:** Capacity losses in 1 M NaPF_6_‐EC:DEC and 1 M NaTFSI‐EC:DEC for the three cycling protocols as shown in Figure 1 and Figure S3b,c, Supporting Information.

Cycling protocols	Capacity loss mechanism	NaPF_6_‐EC:DEC	NaTFSI‐EC:DEC
1	Continuous SEI dissolution and reformation, chemical desodiation and SEI growth, and diffusion‐controlled Na‐trapping	34 ± 2 µAh	28 ± 4 µAh
2	Continuous SEI dissolution and reformation, chemical desodiation and SEI growth, and diffusion‐controlled Na‐trapping	23 ± 3 µAh	24 ± 2 µAh
3	“One‐time” SEI dissolution	8 ± 4 µAh	9 ± 5 µAh

The uncertainties depict the standard deviations based on the data from three replicate cells.

The capacity losses seen for using protocol 3, which mainly should have been due to a “one‐time” SEI dissolution (as no SEI could be reformed at the high open circuit potential) were about 8 to 9 µAh (i.e., 4.4–5 mAh g^−1^). The fact that the capacity losses were similar in 1 M NaPF_6_‐EC:DEC and 1 M NaTFSI‐EC:DEC (i.e., ≈8–9 µAh) for protocol 3 indicates that the SEI dissolution rates were very similar in these two electrolytes. This further suggests that the aging mechanism of SEI dissolution mainly was determined by the employed solvents, rather than the sodium salts. However, it should be emphasized that this conclusion is valid for the electrolyte salts and solvents used in this study. The use of other electrolyte salts and solvents can of course affect the composition and stability of the SEI layer depending on, for example, their reduction potentials and electrolyte solvation structures. The work presented here can further be used to identify and quantify the influence of different aging mechanisms for different electrolytes and negative electrode materials.

The capacity losses measured by protocol 1 were about 34 and 28 µAh for the cells with 1 M NaPF_6_.EC:DEC and 1 M NaTFSI‐EC:DEC, respectively. These capacity losses are quite substantial as they account for almost 20–30% of total capacities obtained from CB electrodes (see Figures S4 and S5, Supporting Information). The differences between the measured capacity losses for the first and third protocols for the two electrolytes were about 26 and 19 µAh (i.e., 14.4 and 10 mAh g^−1^), respectively. These results hence demonstrate that larger capacity losses were seen when the CB electrode was sodiated rather than desodiated. Since the capacity loss at the desodiated state was mainly assigned to SEI dissolution, the increased capacity loss at the sodiated state stemmed from additional aging mechanisms such as chemical desodiation and subsequent SEI growth and sodium trapping in the core of CB particles.

### 2.2 Compositional Changes of the Electrolyte

**Figure 3:**
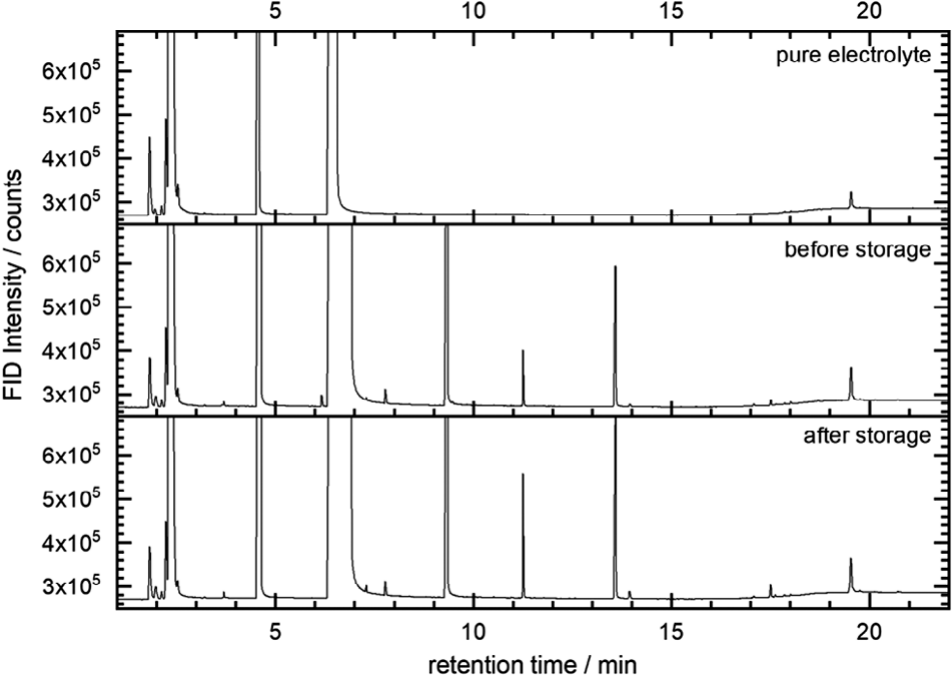
GC analysis (detector: FID) of 1 M NaPF6 in EC:DEC electrolyte as well as the electrolyte extracted prior to and after a 50‐h open circuit pause.

As a result of the dissolution of SEI, the concentration of the SEI species should increase in the electrolyte. Such changes may be negligible if the ratio between the electrolyte volume and the surface area of the electrode is high enough (this is often the case for battery cells made in academic research labs). For commercial cells, where a minimum amount of electrolyte is used, the SEI dissolution can, however, have insignificant influence on the lifetime of a battery. To analyze the changes in the composition of the electrolyte due to SEI dissolution, gas chromatography (GC) coupled with mass spectroscopy (MS) and flame ionization detector (FID) in parallel was used.^[^ ^21^ ^]^ The 1 M NaPF_6_ in EC:DEC from the cells prior to and after an open circuit pause were compared to the pristine electrolyte. The results revealed that both electrolytes from the cycled cells contained numerous reaction and decomposition products. In contrast, the pristine electrolyte only contained both solvents DEC and EC. An overview of the results of the measurements is shown in **Figure** **3** while the different compounds are listed in Table S1, Supporting Information.

**Figure 4:**
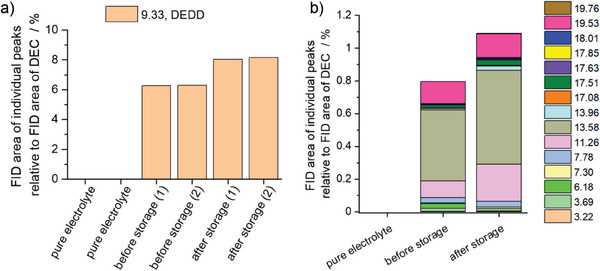
Semiquantitative GC analysis (detector: FID) of the pure electrolyte as well as the extracted electrolyte before and after a 50‐h open circuit pause. a) Diethyl 2,5‐dioxahexanedioate (DEDD) related to DEC at a retention time of 9.33 min (two individual measurements are shown). b) Remaining compounds detected with FID related to the solvent DEC (median values of two individual measurements) are referred to their retention times.

To obtain a semiquantitative analysis, the areas of the observed peaks in the FID chromatogram were determined and related to the DEC area (**Figure** **4**). Here, the DEC content is not expected to differ significantly before storage and after storage. It should be noted that while absolute statements are not possible, conclusions regarding changes in the electrolyte composition before and after the open circuit pause can be made. The results indicate that diethyl 2,5‐dioxahexanedioate (DEDD) as one of the electrolyte decomposition products was present in up to a %‐concentration and that the content increased significantly after the open circuit pause. Additionally, other compounds exhibited increases in their electrolyte contents during the open circuit pause (e.g., compounds at retention times of 7.30, 11.26, 13.58, and 17.51 min, see Figure 4). Therefore, the GC results further support the hypothesis that considerable amounts of SEI species dissolved into the electrolyte during the open circuit pause. Interestingly, the bis(2‐methoxyethyl) ether (diglyme, G2), which was detected in the electrolyte directly after cycling at a retention time of 6.18 min (RI value of 945), disappeared completely after the open circuit pause. This indicates that G2 reacted completely or to a very large extent during the open circuit pause. This could explain the aforementioned results that some of the inserted sodium was chemically desodiated and consumed during the storage time. Additionally, ^1^H‐NMR measurements performed on electrolytes before and after the open circuit pause (Figure S6, Supporting Information) support the findings that the concentration of dissolved SEI compounds in the electrolyte increased during the pause.

**Figure 5:**
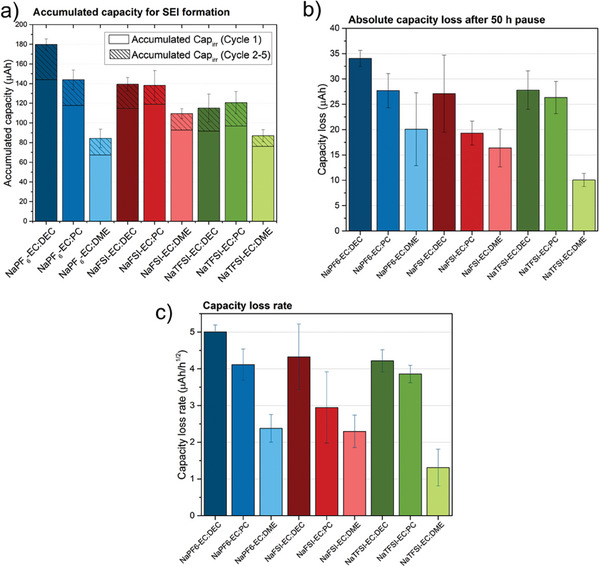
Galvanostatic results for the nine different electrolyte systems. The uncertainties represent the SD based on three replicate cells. a) Accumulated capacity for cycles 1 to 5. b) Absolute capacity loss after a 50‐h pause in each electrolyte system. c) Capacity loss rate as determined from Figure S7, Supporting Information.

Following all the results and discussion above, we further studied the influence of the electrolyte composition on the aforementioned aging mechanisms. This can be helpful in quantifying how much of the total observed capacity losses originated from Na‐trapping. So, nine different non‐aqueous electrolyte systems (see **Table** **2**) were investigated using protocol 1, with pause intervals of 50, 30, 15, and 5 h implemented after five cycles. Three different Na‐salt of sodium hexafluorophosphate (NaPF_6_), sodium bis(fluorosulfonyl)imide (NaFSI) and sodium trifluoromethanesulfonimide (NaTFSI) in three different solvent mixtures in which ethylene carbonate (EC) solvent was mixed with either diethylene carbonate (DEC), propylene carbonate (PC) or 1,2‐dimethoxyethane (DME) in (1:1) volumetric ratio were investigated (see Table 2). Again, the capacity difference between sodiation and desodiation was taken as a measure of irreversible capacity.^[^ ^16^ ^]^ As shown in Figure S4, Supporting Information, the desodiation and sodiation capacities after the first cycle ranged from 50 to 90 µAh (i.e., 28 to 50 mAh g^−1^) per cycle, and the irreversible capacity was almost zero after the first cycle (see Figure S5, Supporting Information). To compare the charge consumption for the first and following cycles in the different electrolyte systems, the irreversible capacities were plotted as a function of the cycle number as seen in **Figure** **5**. The first irreversible charge, which was significantly larger than the rest of the irreversible capacities, corresponds to the SEI formation (see Figure 5a). The highest irreversible first cycle capacity was observed in 1 M NaPF_6_‐EC:DEC while the lowest value was found using 1 M NaTFSI‐EC:DME. The accumulated irreversible capacity for cycles 2 to 5, which was significantly smaller than the first cycle irreversible capacity, showed that the EC:DME electrolytes featured the lowest accumulated irreversible capacities irrespective of the salt used (85 to 110 µAh, corresponding to specific capacities of 47 to 61 mAh/g). The irreversible charge consumption on cycles 2 to 5 can be considered to represent further SEI formation and Na insertion to compensate for the SEI dissolution and Na‐trapping into the carbon particles during initial cycles. Figure 5b shows the absolute capacity losses after a 50 h pause for all the studied electrolyte systems. The largest capacity loss was observed in EC:DEC‐based electrolytes (25–35 µAh, i.e., 14–19 mAh g^−1^, after a 50‐h pause independent from the conducting salt) while the smallest capacity loss was measured in EC:DME‐based electrolytes (10–20 µAh, also independent from the salt). This indicated that the chemistry of the solvent has a higher impact than that of the salt on the measured capacity losses.

**Table 2:** Electrolyte systems investigated in the cells cycled using protocol 1.

Tested electrolyte systems	Solvents	Salts
1 M NaPF_6_ in EC:DEC	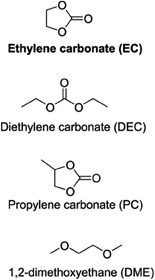	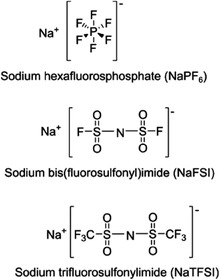
1 M NaPF_6_ in EC:PC		
1 M NaPF_6_ in EC:DME		
1 M NaFSI in EC:DEC		
1 M NaFSI in EC:PC		
1 M NaFSI in EC:DME		
1 M NaTFSI in EC:DEC		
1 M NaTFSI in EC:PC		
1 M NaTFSI in EC:DME		

The capacity loss due to Na‐trapping is independent from the chemistry of the electrolyte solution assuming that the sodiation capacity is not influenced by the electrolyte chemistry. Therefore, the difference between the higher and lowest measured capacity losses after a 50 h pause can hint at how much capacity loss was due to other aging mechanisms than Na‐trapping, that is, SEI dissolution, chemical desodiation, and consequent SEI formation.

The capacity loss was plotted as the function of the square root of the pause time (see Figure S7, Supporting Information) and as a function of the pause time (see Figure S8, Supporting Information) where the former showed a better match with the data. The obtained rate of the capacity loss can be used to compare how fast an electrolyte system undergoes self‐discharge (see Figure 5c). Similar to the capacity losses seen in Figure 5b after a 50‐h pause, the lowest loss rate was found in NaTFSI‐EC:DME (1.3 µAh h^−1/2^_pause,_ i.e., 0.72 mAh g·h^−1/2^_pause_) while the highest capacity loss rate was observed in the NaPF_6_‐EC:DEC electrolyte system (5 µAh h^−1/2^_pause_, i.e., 2.78 mAh g·h^−1/2^_pause_).

**Figure 6:**
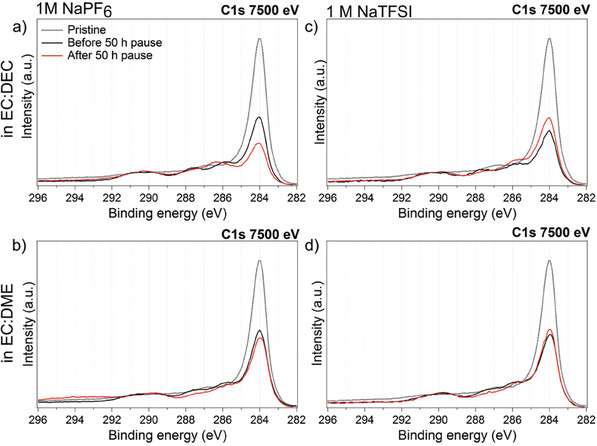
C 1s spectra of pristine and cycled CB electrodes cycled in different electrolytes using a photon energy of 7500 eV.

For the cells with EC:DME‐based electrolytes, featuring the lowest capacity losses during the pause, one expects very little changes in the composition of the SEI during the storage. This means that a dense SEI layer is likely formed which prevents current leakage via SEI and dissolution of SEI species. Similarly, the highest changes in the composition of the SEI during the pause are expected for the cell with the highest irreversible capacity, that is, EC:DEC‐based cells. This was confirmed by synchrotron‐based XPS analysis on the cycled CB electrodes before and after the pause. As shown in **Figure** **6**, the C 1s spectra measured with a photon energy of 7500 eV remain similar before and after the pause for the cells cycled with EC:DME independent of whether NaPF_6_ or NaTFSI was used. On the other hand, the C 1s spectra of the samples cycled in EC:DEC solvent undergoes changes during the pause. This is the case when either NaPF_6_ or NaTFSI salts are used in EC:DEC (the spectra of measured SEI with photon energies of 2500 and 7500 eV and a detailed discussion are presented in Figures S9–S16, Supporting Information). The XPS results therefore confirm the trends seen in the electrochemical results, that EC:DEC was the worst and EC:DME is the best solvent mixture in terms of SEI stability.

## 3 Conclusion

The capacity losses during storage time with no external applied current or potential influence the long‐term lifetime of battery cells. Such losses can be generated from a variety of aging mechanisms and are challenging to probe and quantify. Here, the quantitation of capacity losses due to three different aging mechanisms are discussed, that is, capacity losses due to i) the SEI dissolution and the consequent SEI reformation, ii) chemical desodiation and SEI growth, and iii) diffusion‐controlled trapping of Na into active electrode particles. Three electrolyte salts and three different solvent mixtures have been studied and evaluated with respect to their capacity losses during cycling and open circuit pauses. Using different galvanostatic cycling protocols, the contributions from the aforementioned aging mechanism could be identified successfully. These protocols can readily be used with other electrolyte/cell chemistries to evaluate the capacity losses and therefore the aging in any batteries including alkali metal‐ion batteries accordingly. Furthermore, this study helped to understand the chemistry between electrolyte salt and solvent and its influence on the SEI stability during an open circuit pause. The irreversible capacity due to SEI formation, SEI dissolution, and subsequent SEI reformation and SEI growth depends on both the salt and solvent of the electrolyte. However, the influence of the diffusion‐controlled Na‐trapping should be independent of the electrolyte chemistry given that the sodiation capacity is not influenced by the electrolyte chemistry.

An interdependence between the capacity consumption upon SEI formation and the capacity loss during an open circuit pause was observed, suggesting a correlation between the aging mechanism during cycling and during the pause. Among the tested electrolyte systems, the SEI formed in the EC:DME solvent mixtures exhibited the lowest irreversible capacities during the first five cycles, the lowest absolute capacity losses, and the lowest capacity loss rates during an open circuit pause. The results showed up to 25–35 µAh capacity loss for the worst cells, out of which below 10 µAh could be assigned to SEI dissolution and diffusion‐controlled Na‐trapping while the rest most likely originated from the chemical desodiation and the subsequent SEI growth. Complementary experiments with GC‐MS and ^1^H‐NMR confirmed the influence on the electrolyte matrix due to SEI dissolution and chemical desodiation accompanied by SEI reformation and growth during the pause. The SEI dissolution was highlighted by the increase in DEDD concentration after a 50‐h open circuit pause, whereas the SEI growth during the pause was highlighted by reduced diglyme concentration in the electrolyte. XPS results confirmed that the composition of the SEI remained stable during the pause for the cells with EC:DME‐based electrolytes with the lowest capacity loss, while that changed for the cell with EC:DEC‐based electrolytes with the higher capacity loss. Overall, this work gives new insight regarding how to evaluate the SEI dynamics and how to probe and quantify capacity losses related variety of aging mechanisms electrochemically and spectroscopically. Accordingly, the results are highly relevant both in scientific terms and for industrial applications. The conclusions of this study are of course valid for the electrolyte solvents and salts investigated here as other electrolyte solvents and salts with, for example, different reduction potentials and solvation structure could influence the SEI composition and stability as well as the influence of the aging mechanisms discussed here. The methodology presented in this study can be used to study aging effects using different electrolyte salts and solvents.

## 4 Experimental Section

### Cell Assembly

All experiments were conducted in an argon‐filled glovebox (O_2_ < 1 ppm, H_2_O < 1 ppm). All the used solvents, ethylene carbonate (EC, Gotion), diethylene carbonate (DEC, BASF), propylene carbonate (PC, Gotion), and 1,2‐dimethoxyethane (DME, BASF) were filtered through a 0.2 µm nylon membrane syringe filter (VWR). The solvent mixtures EC:DEC, EC:PC, and EC:DME were all prepared with volume ratio of 1:1. The electrolyte salts sodium trifluoromethane sulfonimide (NaTFSI, Aldrich) and sodium hexafluorophosphate (NaPF_6_, Stella) were vacuum dried for 24 h at 120 °C, whereas the thermally less stable salt sodium bis(fluorosulfonyl)imide (NaFSI, Solvonic) was dried at 60 °C for 48 h under vacuum. Each salt was added to the three electrolyte solvent mixtures to a concentration of 1 M (see Table 1). For the CB electrodes, a mixture of 0.9 g of CB (TIMCAL ENASCO 250P), 0.1 g of sodium carboxymethyl cellulose (CMC, Merck), and 10 ml distilled water was ball‐milled for 1 h at 600 rpm. The slurry was then cast on carbon‐coated aluminum foil with a thickness of 200 µm and dried at 100 °C. Discs with a diameter of 10 mm were then punched out and vacuum dried at 120 °C for 12 h in the argon‐filled glovebox. The average weight of the 10 measured electrodes was 2 ± 0.01 mg. The galvanostatic cycling tests were carried out with Na‐half cells. These contained a CB working electrode (10 mm diameter), a β‐alumina discs (Ionotec; 1 mm thickness; 20 mm diameter) serving as the separator, and a Na‐metal electrode on aluminum foil (14 mm diameter), all assembled in a pouch cell. An illustration of the cell setup is shown in Figure 1c. The electrolyte volume was 150 µL unless stated otherwise. For the comparison between the β‐alumina and Solupor separators, cells were also assembled containing Solupor Lydall – 3PO7A separators instead of β‐alumina separators. In this study, the terms discharge and charge corresponded to sodiation (reduction) and desodiation (oxidation) of the CB electrodes.

### Electrochemical Testing

The galvanostatic experiments were carried out with a Novonix high‐precision cycler system at 30 °C. The cells were cycled from 0.1 to 2.0 V versus Na^+^/Na with a constant current of 50 µA. The cut‐off potential was set to 0.1 V, to avoid underpotential deposition of sodium.^[^ ^22^, ^23^ ^]^ After five cycles, open circuit pauses of 50, 30, 15, and 5 h were applied after stopping at 0.1 or 2.0 V, to study the sodiated and desodiated states of CB. The cycling program is shown in Figures 1b and 2a.

### Characterization XPS

The X‐ray photoelectron (XPS) measurements were conducted at the synchrotron facility Deutsches‐Elektronen‐Synchrotron (DESY) in Hamburg, Germany. The SEI layer formed in six different electrolyte systems, namely 1 M of NaPF_6_ and NaTFSI in EC:DEC, EC:PC, and EC:DME were studied. Two cells were cycled five times for each electrolyte mixture, whereas one was stopped immediately after five cycles at 0.1 V (Before the pause sample) and one after finishing a pause of 50 h (After the pause sample). The cells were then disassembled in the glovebox and the CB electrodes were washed with 0.5 ml dimethyl carbonate (DMC, Sigma). The electrodes were sealed under vacuum for transportation to the synchrotron facility. At DESY, the samples were measured at photon energies of 2500 and 7500 eV. Due to the major presence of sp^[^ ^2^ ^]^ carbon bonds in CB, all spectra were normalized to 284.0 eV and by the cross‐section of the particular core level and the inelastic mean free path (IMFP). With this, the elemental composition could be analyzed based on the relative intensities of the XPS spectra.^[^ ^24^, ^25^ ^]^

### Characterization GC‐MS and ^1^H‐NMR

In addition to the normal cell setup as mentioned before, one sheet of Solupor separator was placed between the carbon black electrode and the β‐alumina separator. After reaching the desired state (cycled five times before or after a 50‐h pause), the separator was removed. For the samples taken before and after the pause, the separators were soaked in 1.5 ml dichloromethane (CH_2_Cl_2_) for 30 min (shaken by hand), after which the liquid was taken in a centrifugal vial, centrifuged, liquid taken in a GC vial, measured (two times injected each). For the measurement, a volume of 40 µl of 1 M NaPF_6_ in EC:DEC electrolyte was diluted in CH_2_Cl_2_ for 15 min and then centrifuged. For the 1H‐NMR measurements either 50 µl pristine electrolyte or the separators of the cycled cells (before the pause, after a 5‐ h and a 50‐h pause) were added to 0.6 ml deuterated dimethyl sulfoxide (d_6_‐DMSO). Gas chromatography experiments were performed with a Clarus 690 GC from PerkinElmer Inc. (Waltham, USA) which was equipped with an autosampler, a flame ionization detector (FID), and an MS detector (SQ 8T). Turbomass 6.1.2 and OriginLab 2021b software packages were used for data acquisition as well as data analysis. Briefly, the following parameters were used during the GC measurement: Gas: He 6.0 (Air Liquide), H_2_ gas from hydrogen generator (PG+160, Vici DBS), Air (Air Liquide); Column: Optima 5MS, 30 m length × 0.25 mm interior diameter, 0.5 µm film thickness; Parameters during injection: the split flow of 20 ml min^−1^, the inlet temperature of 250 °C, 0.5 µl injection volume, 175 kPa initial pressure, pressure controlled mode, oven temperature 40 °C; Oven and pressure parameters: 40 °C for 1.5 min, heating at 20 °C min^−1^ up to 320 °C; pressure starting from 175 kPa for 2 min, increase at 7.8 kPa min^−1^ up to 300 kPa; MS setup: filament voltage of 70 kV, ion source temperature of 200 °C, MS transfer line temperature of 200 °C; FID setup: 450 ml min^−1^ for synthetic air, 45 ml min^−1^ for hydrogen gas FID temperature of 280 °C. The gas flow was split by a SilFlow GC Capillary Column 3‐port Splitter after the separation column (to MS and FID detector). The MS was used in the scan mode with a scanned range from 33 to 350 u. The signals from the FID were used for determining the peak area. Impurities in the electrolyte solvents were analyzed, when possible, based on NIST search (EI fragmentation match) as well as measuring the pure compounds. All peaks were analyzed and referred to RI values based on n‐alkanes.

## Conflict of Interest

The authors declare no conflict of interest.

## Author Contributions

L.A.M. wrote the manuscript. L.A.M. and A.B. carried out electrochemical and synchrotron‐based XPS measurements and evaluation. A.H. conducted the GC investigations and evaluated the GC data. R.Y. and L.N. were involved in experimental planning and project discussions. All authors helped with data interpretation.

## Supporting information

Supporting Information

## Data Availability

The data that support the findings of this study are available from the corresponding author upon reasonable request.
