# Supplementary material for: Fundamental Understanding and Quantification of Capacity Losses Involving the Negative Electrode in Sodium‐Ion Batteries
Source: Adv Sci (Weinh). 2023 Dec 7;11(6):2306771. doi: 10.1002/advs.202306771 (PMC10853709; doi:10.1002/advs.202306771)
Supplement: Supplementary file 1 — Supporting Information [file ADVS-11-2306771-s001.pdf]

ADVANCED SCIENCE
Open Access

Supporting Information

for *Adv. Sci.*, DOI 10.1002/advs.202306771

Fundamental Understanding and Quantification of Capacity Losses Involving the Negative Electrode in Sodium-Ion Batteries

*Le Anh Ma, Alexander Buckel, Andreas Hofmann, Leif Nyholm and Reza Younesi**

Supporting information

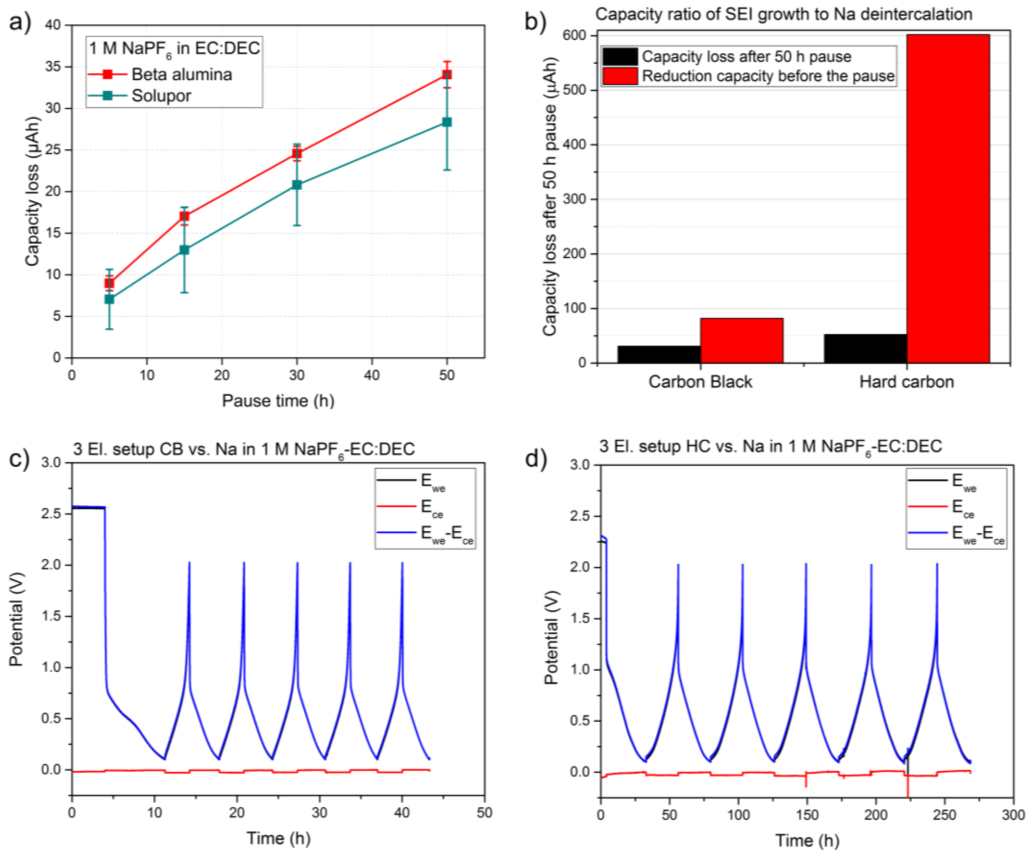

Figure S1. Galvanostatic cycling results comparing β-alumina with Solupor® separators and carbon black with hard carbon electrodes. a) Absolute capacity loss for the two different separators (red: β-alumina, green: Solupor®) as a function of the open circuit pause time. The cell with Solupor® separator resulted in smaller capacity losses which can be explained by the electrolyte reduction species, formed at the Na-metal electrode, saturating the electrolyte system, and hindering additional SEI dissolution. With the Na-conductive β-alumina separator, the influence of the species formed at the Na-metal on the carbon black working electrode can be decreased and the electrolyte systems can hence be studied without being affected by the species formed at Na-metal electrode. b) “Signal-to-noise” ratio: Hard carbon and carbon black working electrodes

were cycled five times, paused for 50 hours, and cycled subsequently in a three-electrode setup with β-alumina separator in 1 M $NaPF_6$-EC:DEC. The ratio between reduction capacity before the pause and the absolute capacity loss due to SEI dissolution/reformation as well as Na-trapping after the 50-hour pause was significantly higher with the hard carbon (602 compared to 52 μAh) than with the carbon black electrodes (82 compared to 31 μAh). Hence, carbon black electrodes are more suitable to study SEI formation and growth due to its better "signal-to-noise" ratio. c) Thee-electrode setup with carbon black working electrode and β-alumina separator in 1 M $NaPF_6$-EC:DEC at a constant current of 25 μA between 0.1 and 2.0 *vs.* Na^+/Na. The results show that the Na-metal counter electrode in this setup with β-alumina is stable during the tested voltage window over a number of cycles. d) Three-electrode setup with hard carbon and β-alumina separator in 1 M $NaPF_6$-EC:DEC at a constant current of 25 μA between 0.1 and 2.0 *vs.* Na^+/Na. The results show that the setup with β-alumina could also be suitable for tests on hard carbon electrodes.

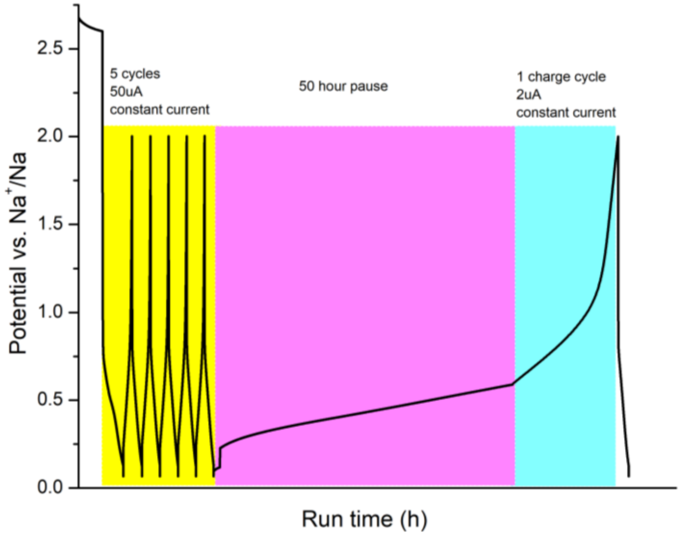

Figure S2. Galvanostatic cycling with 50 hours pause time. The applied current is 50 μA before the pause, but it is reduced to 2 μA for the desodiation (oxidation) after the pause. The results indicates that a larger amount of Na, which is redistributed and trapped during the pause, can be extracted when the current is lowered.

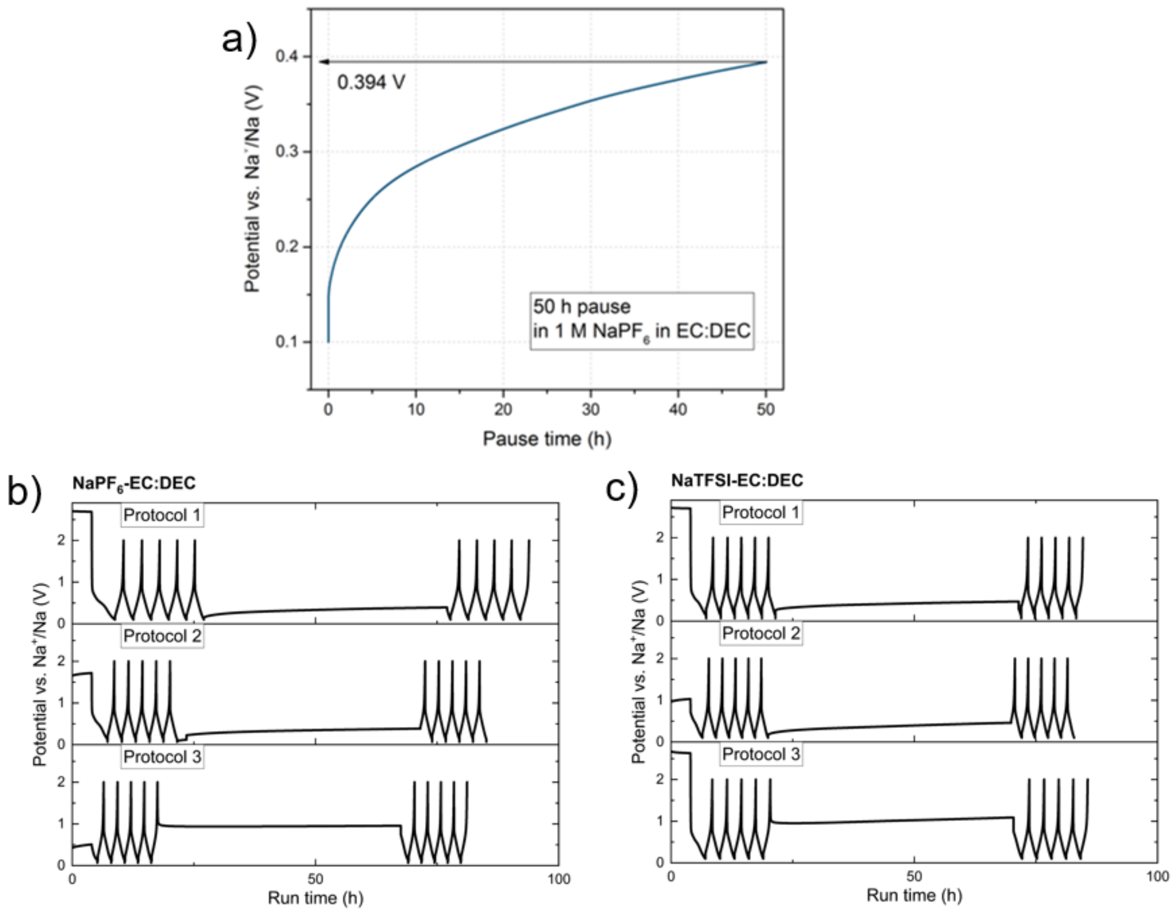

Figure S3. a) Galvanostatic cycling results showing the potential of the CB electrode as a function of the open circuit pause time. The potential increase during the pause can be ascribed to variety of charge loss mechanisms discussed in the manuscript. b) Cycling protocol 1-3 in $NaPF_6$ in EC:DEC. c) Cycling protocol 1-3 in NaTFSI in EC:DEC.

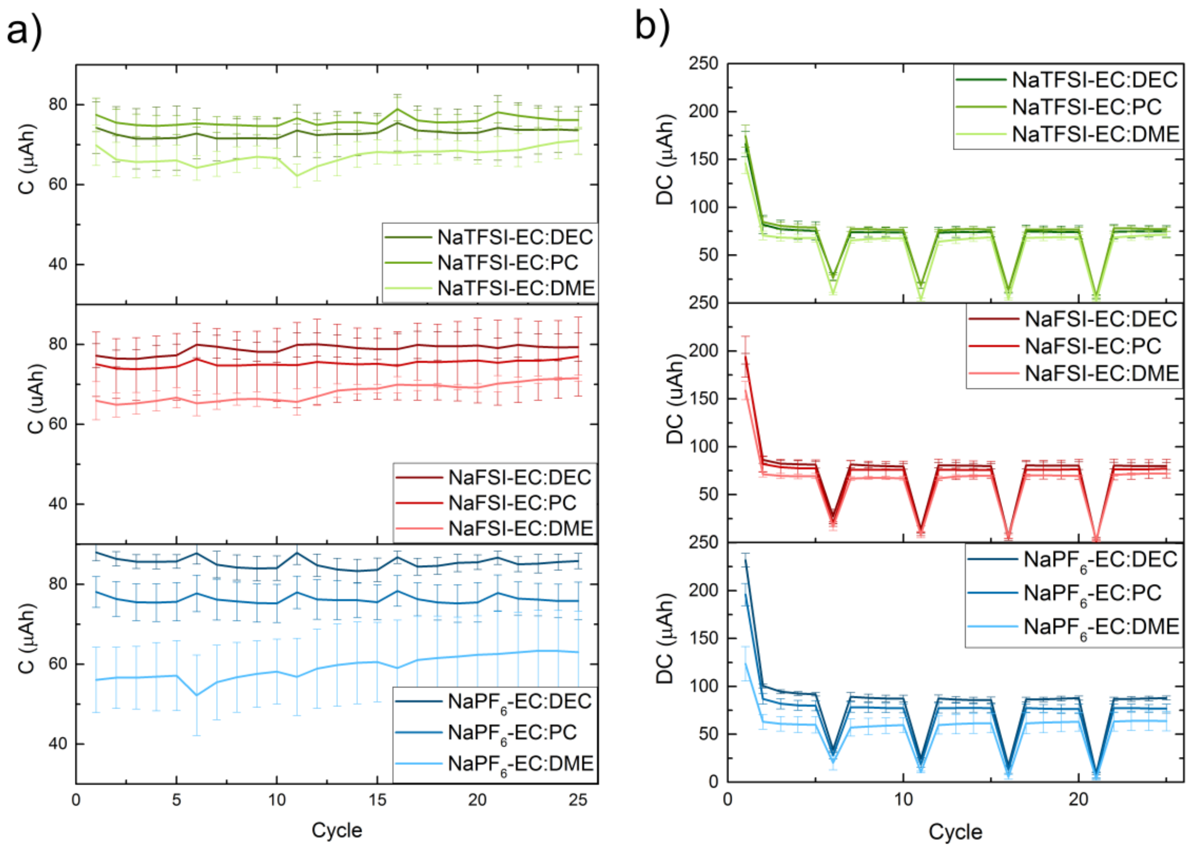

Figure S4. Galvanostatic cycling results. a) Obtained charge (i.e., desodiation) capacities in different electrolyte systems plotted as a function of the cycle number for the different electrolyte systems (green: NaTFSI, blue: $NaPF_6$). b) Obtained discharge (i.e., sodiation) capacities as a function of the cycle as in (a) but with added pause times. Note that the decreases on cycles 6, 11, 16 and 21, represent the capacity losses after an open circuit pause (50, 30, 15 and 5 h).

Compared to the EC:DEC and EC:PC systems with charge (*i.e.*, desodiation) capacities of around 70 to 90 µAh, the EC:DME systems exhibited the lowest charge capacities between 50 to 70 µAh. In EC:DME less Na could hence be extracted from the CB during the charge which most likely was due to the fact that less Na was stored in the CB electrode during the preceding discharge (i.e., sodiation) step. Disregarding the first

cycle and the open circuit pause containing cycles, the discharge capacities ranged between 50 to 100 μAh for all tested electrolyte systems. These values were similar to the corresponding charge capacities. The first cycle discharge capacity was significantly higher than those of the subsequent cycles, indicating a charge consumption due to SEI formation and Na-trapping. For the EC:DEC and EC:PC solvent systems, the discharge capacities were generally higher than for the EC:DME systems. This effect was most significant when using $NaPF_6$. In general, the charge and discharge capacities in $NaPF_6$-based electrolytes showed larger discrepancies for the different solvents than those seen when using NaTFSI.

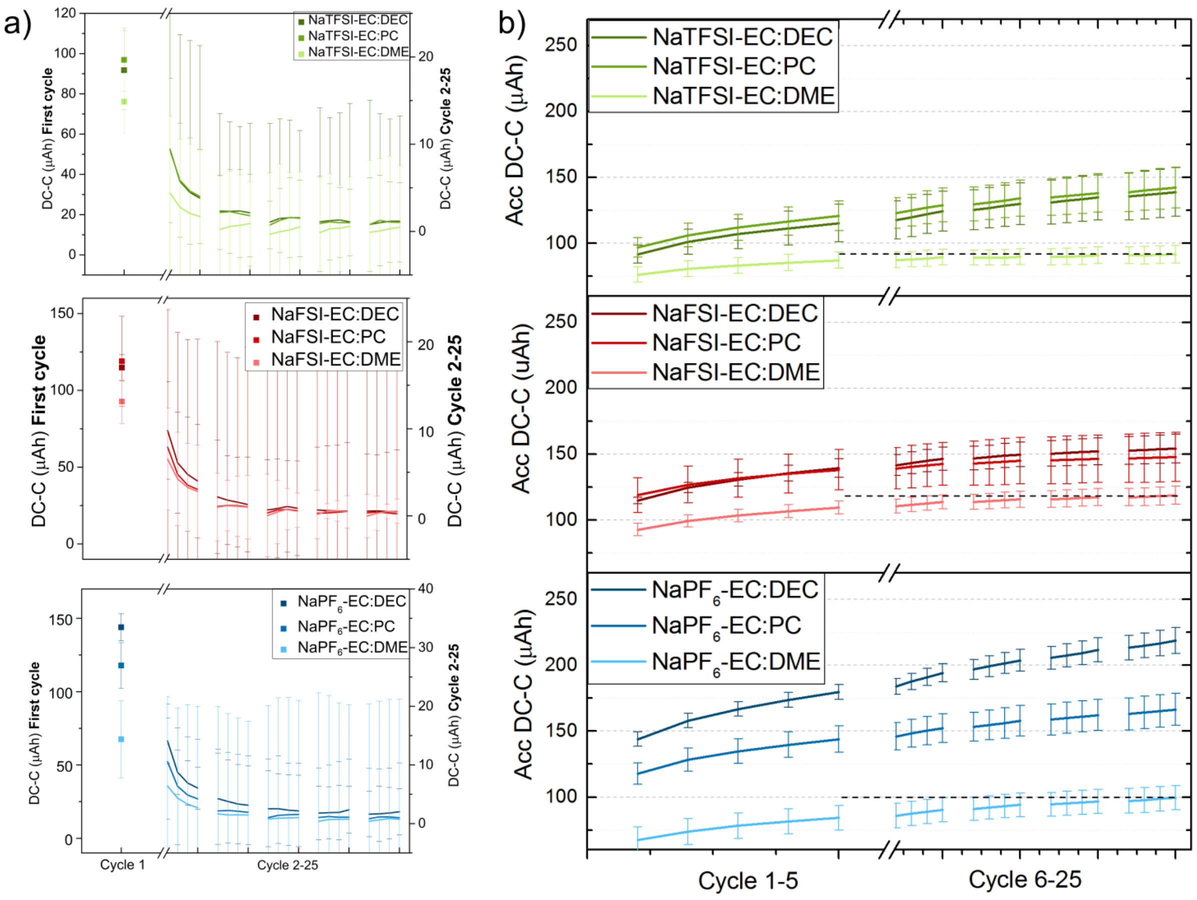

Figure S5. Galvanostatic cycling results with implemented pauses of 50, 30, 15 and 5 hours. The error bars represent the standard deviation of three replicate cells. a) Difference between the discharge and charge capacities plotted against the cycle number for the different salt systems. The left-hand y-axes correspond to the first cycle results, whereas the right-hand y-axes correspond to the results for the subsequent cycles 2 – 25. b) Accumulated difference between the discharge and charge capacities as a function of the number of cycles for the different salt systems. For clarity, the cycles containing an open circuit pause were disregarded in these plots.

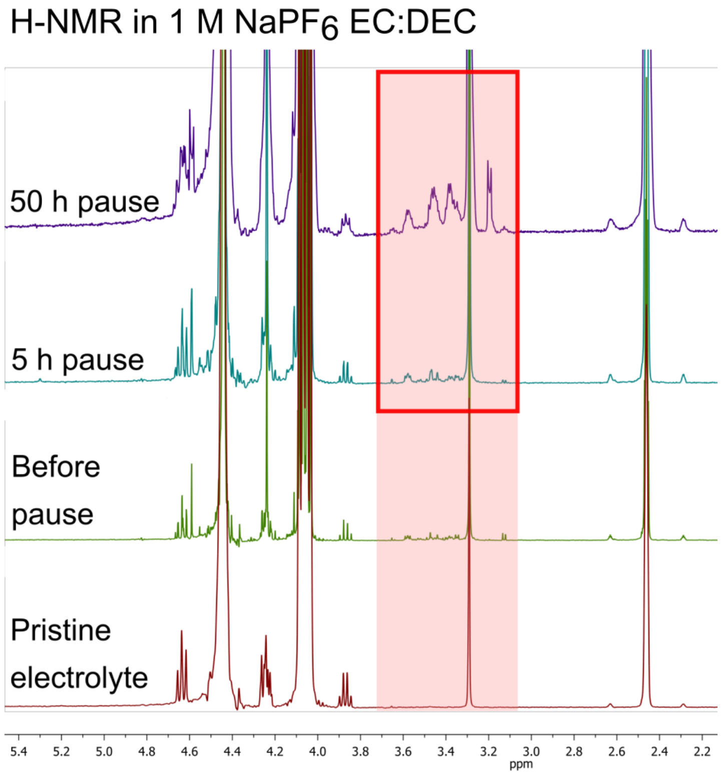

Figure S6. Proton nuclear magnetic resonance (^{1}H-NMR) measurements of pristine electrolyte and cycled (for five times) electrolytes before pause, after a 5-hour and a 50-hour pause. The red box shows the additional peaks after longer pause times, implying dissolved SEI compounds in the electrolyte matrix.

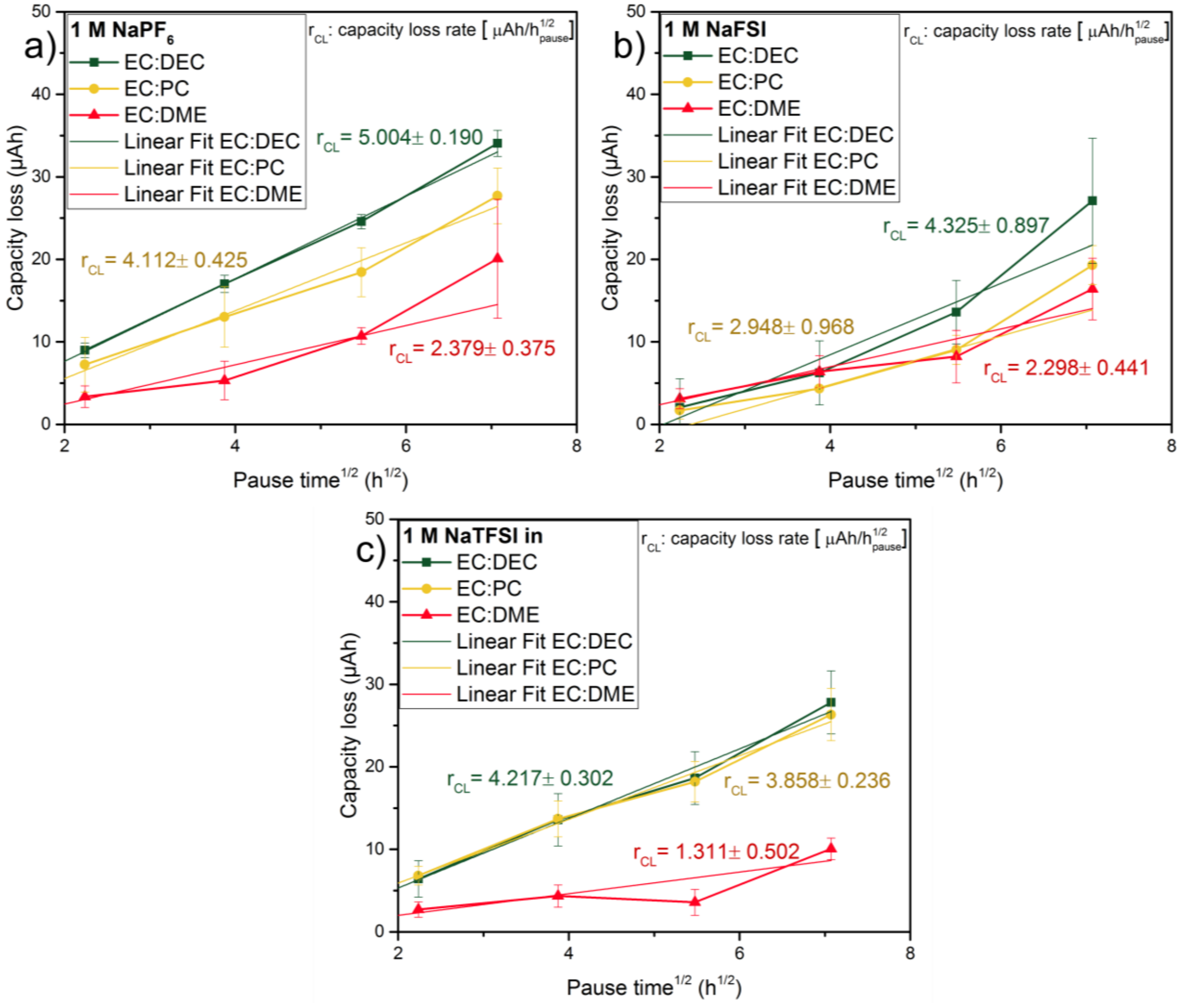

Figure S7. Protocol 1 capacity losses plotted as the function of the square root of the pause times of 50, 30, 15 and 5 hours for a) $NaPF_6$, b) NaFSI and c) NaTFSI each in the solvent mixtures EC:DEC, EC:PC and EC:DME to estimate the rate of self-discharge over pause time. The slope of each curve represents the capacity loss rate during the open circuit pause. For all three electrolyte salt systems, the use of the EC:DME solvent mixture resulted in lower capacity loss rates.

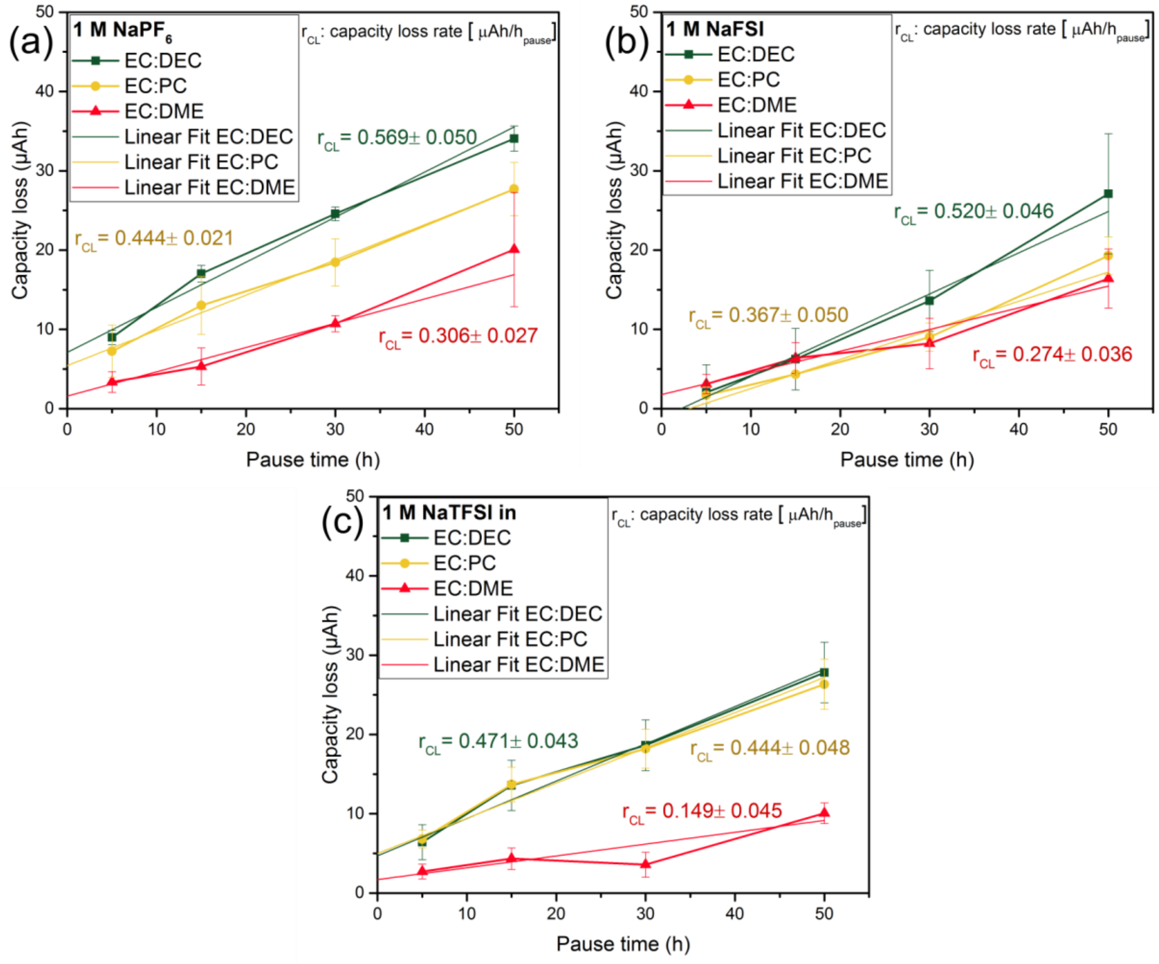

Figure S8. Protocol 1 capacity losses plotted as the function of the open circuit pause times of 50, 30, 15 and 5 hours for a) $NaPF_6$, b) NaFSI and c) NaTFSI each in the solvent mixtures EC:DEC, EC:PC and EC:DME to estimate the rate of self-discharge over pause time. The slope of each curve represents the capacity loss rate during the open circuit pause. For all three electrolyte salt systems, the use of the EC:DME solvent mixture resulted in lower capacity loss rates.

X-ray photoelectron studies of the SEI coated surfaces. To understand how the SEI formation and the SEI composition and stability depend on the electrolyte, synchrotron-based X-ray photoelectron spectroscopy (XPS) experiments with photon energies of 2500 eV and 7500 eV were conducted. The composition SEI formed in electrolyte solutions with either $NaPF_6$ or NaTFSI in all three solvent mixtures were measured. The discussion below highlights changes in the composition of SEI before and after the pause, however, it should be noted that XPS spectra cannot provide quantitative measurement of capacity losses, and thus electrochemical tests should be taken into account to calculate capacity losses. Also, we think the XPS spectra measured with photon energy of 7500 eV provide more reliable results than the spectra measured with photon energy of 2500 eV, because the conclusion made from the spectra measured with photon energy of 7500 are in agreement with conclusion made from electrochemical data regarding the stability of SEI. The higher photon energy with deeper probe provide more reliable information on composition of SEI as the top surface of SEI could be altered during the sample preparation (i.e. washing electrodes with 0.5 ml of DMC).

Figures S6 and S7 show C 1s spectra of the pristine CB electrode and CB electrode before and after a 50-hour pause cycled in the $NaPF_6$ and NaTFSI systems, respectively. For all surface sensitive C 1s spectra, the relative intensity of the C-C peak at 284.0 eV, which corresponds to the

pristine electrode, decreased and became less visible after the cycling. While this confirmed the formation of a SEI layer on the surface electrodes, the C-C peak was still detectable. This indicated that the SEI was thinner than about 20 nm (this is a rough estimation of the probing depth for a photon energy of 2500 eV). The bulk sensitive spectra show a strong C-C peak at 284.0 eV, correlating to a higher probing depth and hence more signal from the bulk CB electrode material. In general, the bulk sensitive C 1s spectra were similar for all electrolyte systems. The C 1s spectra of all the six samples showed the presence of C-O, C-O_2, and -CO_3 species in the SEI via pekas at binding energies of 286 eV, ~287.7 eV, and 290.0 eV.[2015,21,22] For both electrolyte salt systems, NaF was only present in the bulk SEI and not on the surface (see Figure S9), which is coherent with reported SEI models.[5,6,23,24] In Li-based systems, it has been shown that more organic and semi-carbonate components are formed at the SEI/electrolyte interphase, whereas more inorganic species such as Li_2O and LiF are found at the electrode/SEI interface.[5,6,23] Such microphase construction of inorganic and organic SEI layers in Li-based electrolyte is therefore also observed for these Na-based electrolytes.[7] Overall, the XPS spectra obtained for all the samples prior to the extended pause (all the black color spectra in Figures S7 to S12) display similar features.

Comparing the XPS results for the $NaPF_6$ and NaTFSI electrolytes, it is seen that the SEI compositions did not change significantly after the open circuit pause which could be the result of the equal and/or very little dissolution of SEI species (see Figure S13). With regards of the surface-sensitive probing depth of less than 20 nm and the slight peak intensity change corresponding to the CB electrode at 284.0 eV (see Figure S7), the extent SEI dissolution must be very small. This is in agreement with the galvanostatic results obtained with Protocol 3 for $NaPF_6$-EC:DEC and NaTFSI-EC:DEC in Table 2. The $NaPF_6$-EC:DME electrolyte featured the most significant compositional changes in the SEI after the 50-hour pause (see Figure S13a). Generally, the SEI formed in $NaPF_6$ electrolytes mainly show carbon-oxygen species on the surface corresponding to organic and carbonate components, whereas the N and S contents were larger in NaTFSI due to the electrolyte salt. Compared to $NaPF_6$-EC:DEC and –EC:PC, the alkoxides, carboxyl and carbonate components in the SEI of $NaPF_6$-EC:DME decreased after the pause, while the intensities for NaF and Na_2O increased. The carboxyl and carbonate components in the SEI of NaTFSI-EC:DME also underwent dissolution during the pause, yet to a smaller extent compared to that seen for $NaPF_6$. In comparison to the surface, the bulk SEI in NaTFSI electrolytes showed less electrolyte salt decomposition products, whereas for the $NaPF_6$ systems, the bulk SEI contained more salt decomposition species resulting in higher F content (see Figure S13).

For both $NaPF_6$ and NaTFSI salt, the highest capacity loss was observed for EC:DEC (see Figure 5) while the best solvent mixture, *i.e.* EC:DME, showed different trends before and after the pause. The corresponding peak of the CB electrode at 284.0 eV decreased for $NaPF_6$-EC:DEC and -EC:DME (see Figures 6a and b), whereas for NaTFSI-EC:DEC and –EC:DME it increased (see Figures 6c and d). This means that the SEI in $NaPF_6$-EC:DEC and -EC:DME was growing in thickness during the pause, which indicates electrolyte reduction by the reduced carbon and/or gradual saturation of the electrolyte during cycling preventing more dissolution. In NaTFSI-EC:DEC and -EC:DME the SEI was, on the other the hand, dissolving (approx. less than 2 nm) durinng the 50-hour pause. Such SEI growth during a pause has previously been reported for Li-cells containing carbonaceous electrodes and $LiPF_6$ based electrolytes.[25–27] Similar to $LiPF_6$, the SEI growth during a pause in $NaPF_6$-EC:DEC and –EC:DME could stem from the previously sodiated carbon reducing more electrolyte, forming additional SEI species. Moreover, the increase in the SEI thickness by a few nanometers in the $NaPF_6$ systems was more pronounced with EC:DEC than EC:DME, implying that the SEI in $NaPF_6$-EC:DEC was more unstable than in EC:DME and underwent larger changes (see Figures 6a and b) in agreement with the electrochemical results in Figure 5. Similarly, C-C peak increase in the NaTFSI systems, corresponding to SEI dissolution, was more enhanced in EC:DEC than in EC:DME (see Figures 6c and d). This is also in agreement with the electrochemical results in Figure 5.

The fact that different core levels (C 1s, O 1s, F 1s, P 2p, and Na 1s) display different trends of changes in the tested electrolytes indicate that different species formed in these electrolytes are prone to dissolution in different extend. For example, F 1s spectra measured with photon energy of 7500 eV for electrolytes based on ED:DEC show clear changes in the composition of SEI before and after the pause when NaTFSI salt was used, but show no major changes when $NaPF_6$ was used. In opposite, for the electrolyte based on EC:DME, the spectra measured with photon energy of 7500 eV show minor changes when NaTFSI was used while clear changed was observed when $NaPF_6$ was used.

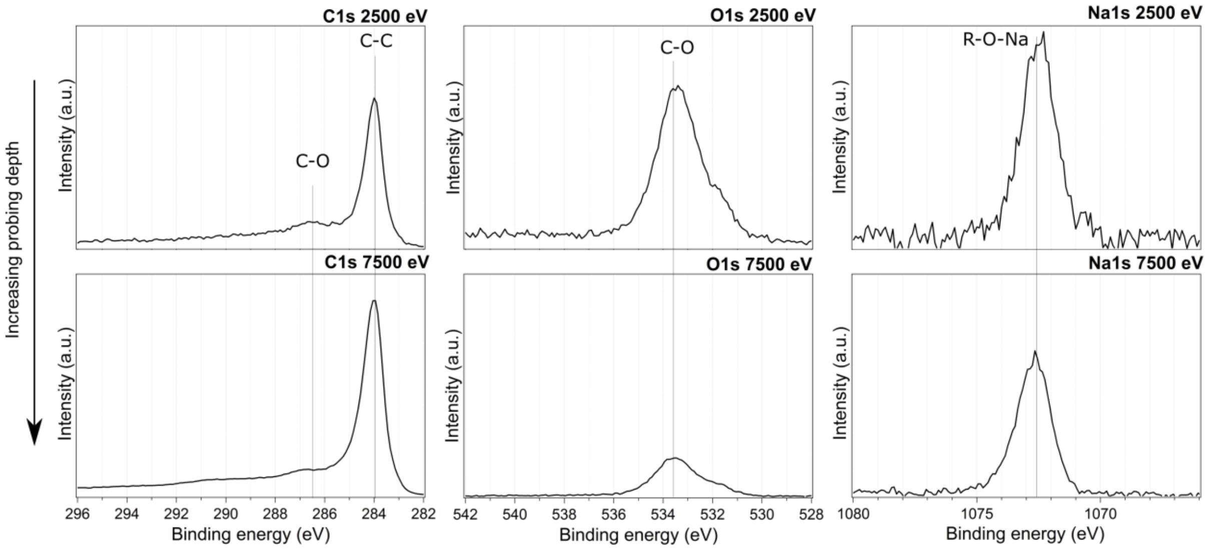

Figure S9. C 1s, O 1s and Na 1s XPS core levels carbon black electrode obtained using different photon energies of 2500 eV and 7500 eV. The peaks correspond to the expected components of the active carbon black material and the binder, *i.e.*, Na-carboxymethyl cellulose (Na-CMC).

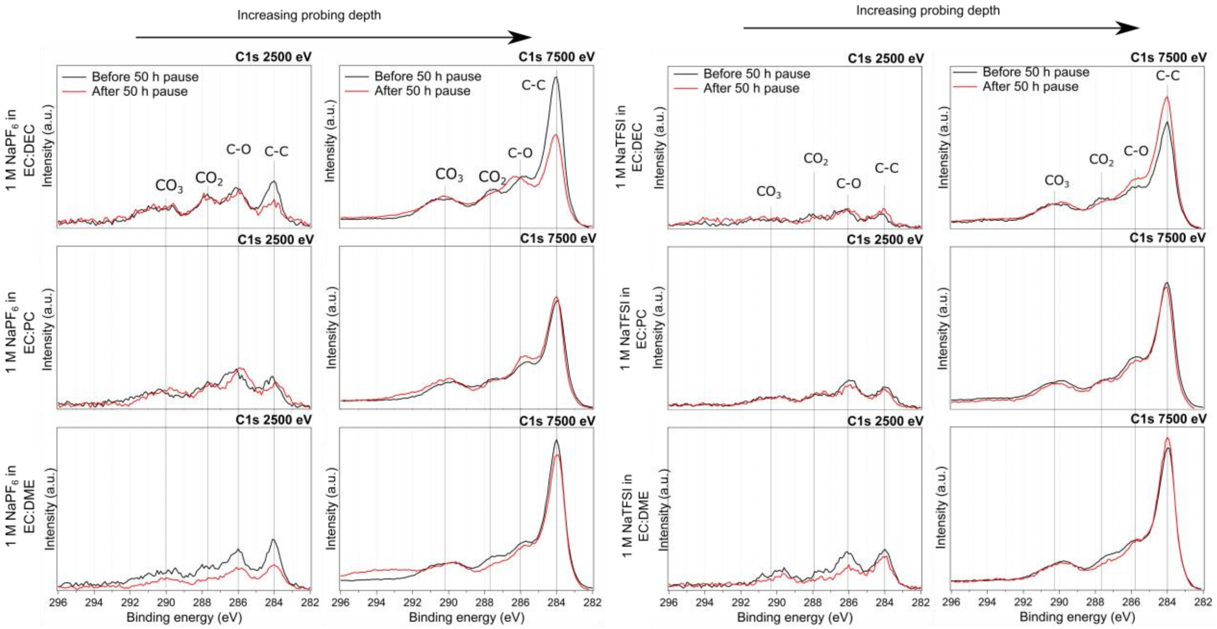

Figure S10. Surface and bulk sensitive XPS results with C 1s spectra of the SEI formed in $NaPF_6$ (left images) in EC:DEC, EC:PC and EC:DME and in NaTFSI (right images) in EC:DEC, EC:PC and EC:DME.

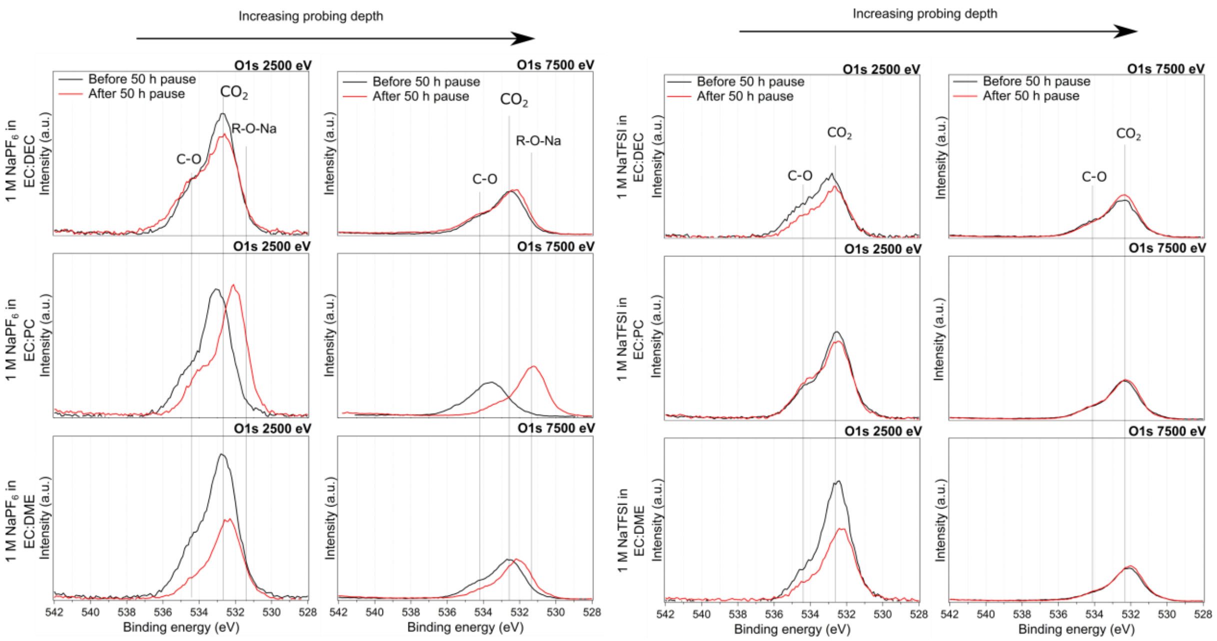

Figure S11. Surface and bulk sensitive XPS results with O 1s spectra of SEI formed in $NaPF_6$ (left images) in EC:DEC, EC:PC and EC:DME and in NaTFSI (right images) in EC:DEC, EC:PC and EC:DME.

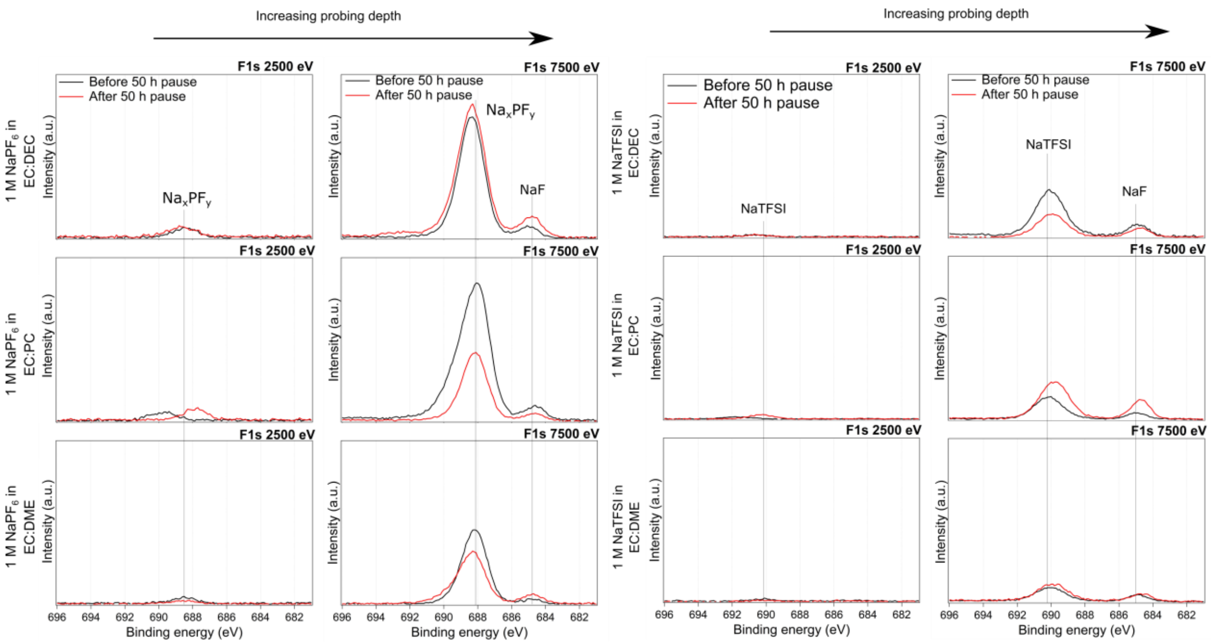

Figure S12. Surface and bulk sensitive XPS results with F 1s spectra of SEI formed in $NaPF_6$ (left images) in EC:DEC, EC:PC and EC:DME and in NaTFSI (right images) in EC:DEC, EC:PC and EC:DME.

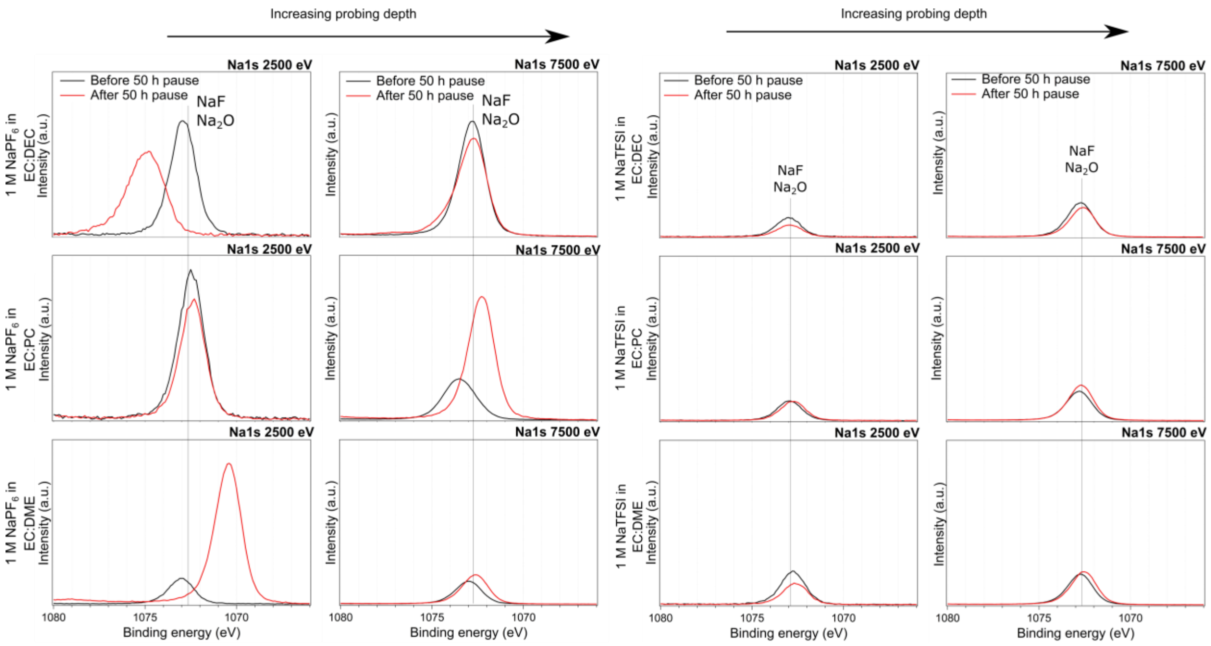

Figure S13. Surface and bulk sensitive XPS results with Na 1s spectra of SEI formed in $NaPF_6$ (left images) in EC:DEC, EC:PC and EC:DME and in NaTFSI (right images) in EC:DEC, EC:PC and EC:DME.

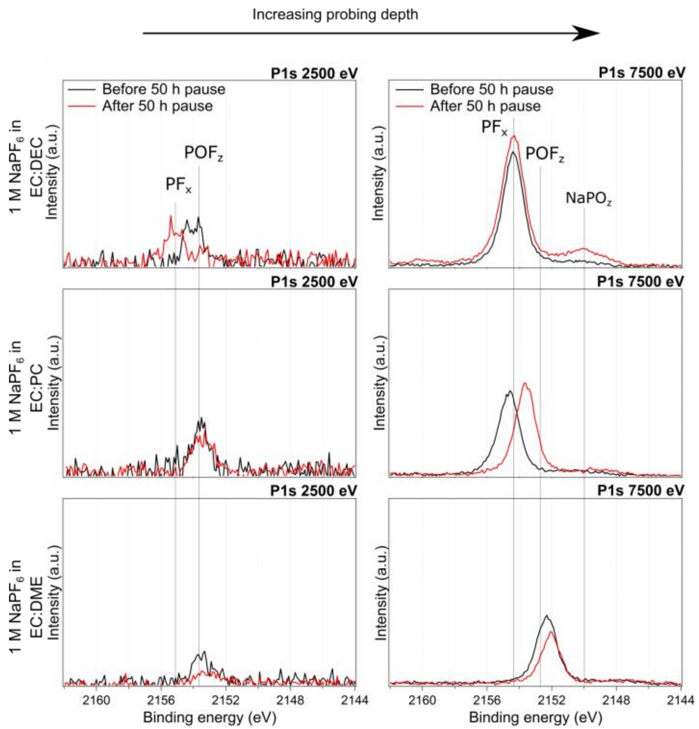

Figure S14. Surface and bulk sensitive XPS results with P 1s spectra of SEI formed in $NaPF_6$ in EC:DEC, EC:PC and EC:DME.

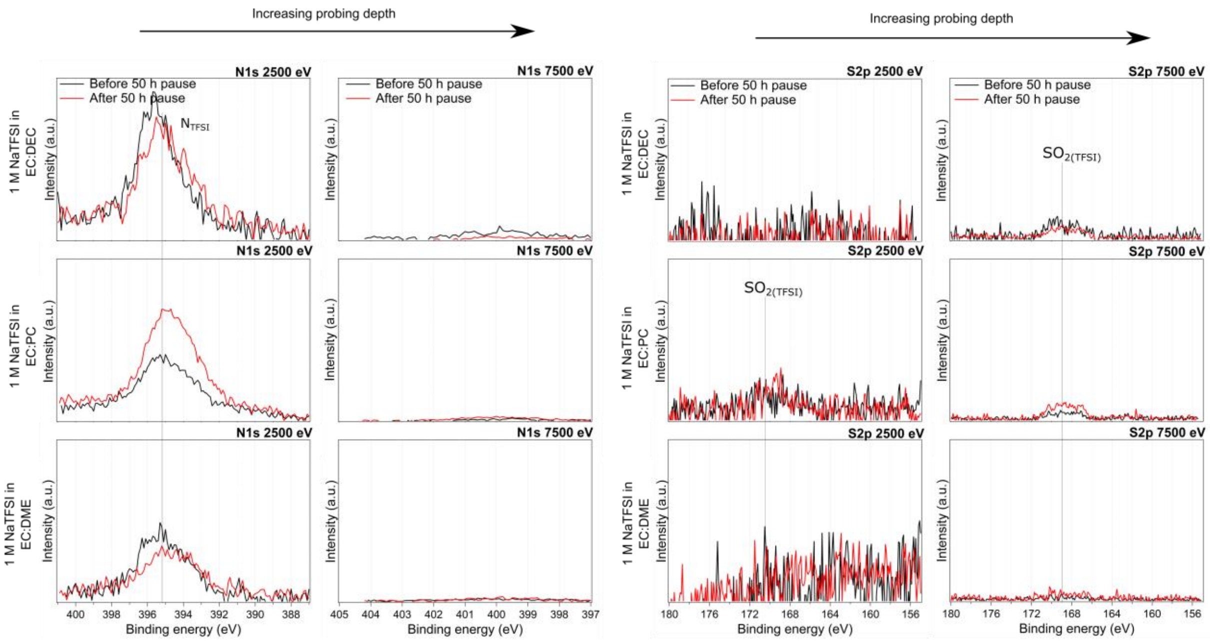

Figure S15. Surface and bulk sensitive XPS results with N 1s (left images) and S 2p spectra (right images) of SEI formed in NaTFSI in EC:DEC, EC:PC and EC:DME.

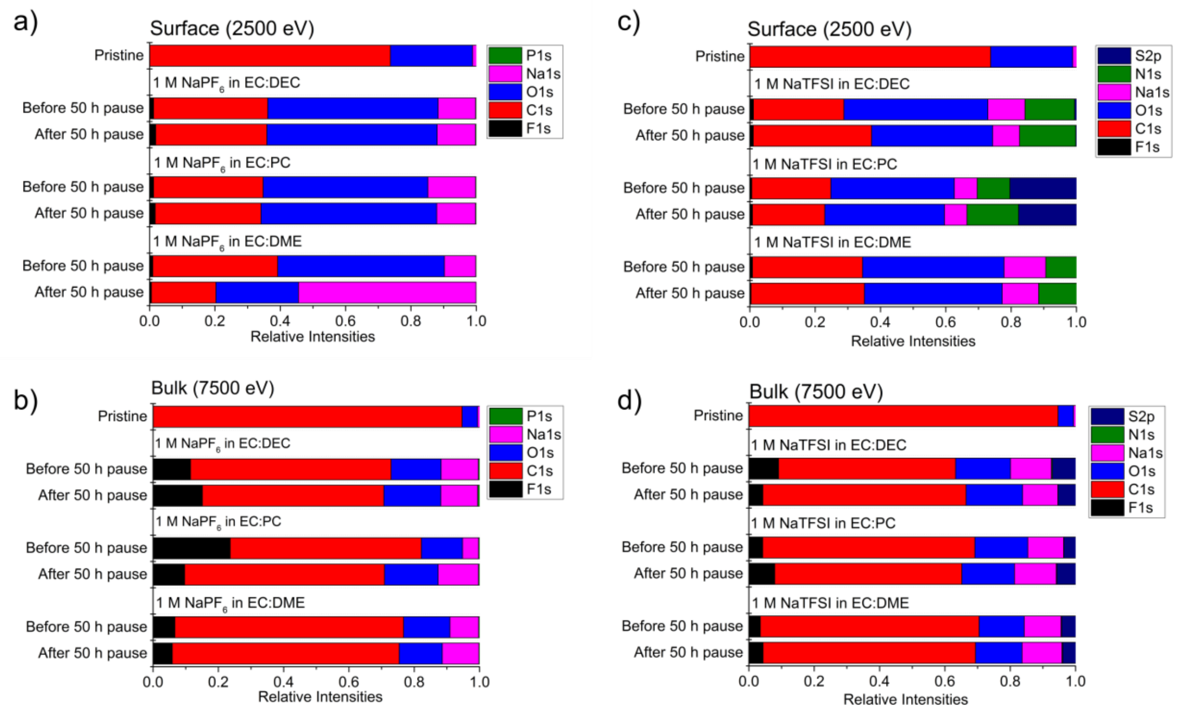

Figure S16. Elemental compositions of the SEI at the surface and in the bulk for all nine tested electrolyte systems based on the XPS spectra shown in Figures S6-12.

Table S1. Compounds found in the pure electrolyte, the extracted electrolyte before storage and the extracted electrolyte after 50 hours under open circuit conditions.

			RT [a] (FID)	RT [a] (MS)	Verified [b]	RI[c]	RI[d]	RI[e]	NIST R. match	*m/z* in descending intensity order					
Name	Abbr.	CAS	[min, Peak max.]	[min, onset]					1000 max.						
**Pure electrolyte**															

Diethyl carbonate	DEC	105-58-8	4.58	4.64	yes	781	781	767	892	45	91	63	43	59	
Ethylene carbonate	EC	96-49-1	6.54	6.43	yes	960	960	(814)		88	43	44	58	42	
**Electrolyte before storage**															
POF_3		13478-20-1		1.84	no				984	104	85	69	50		
Ethyl methyl carbonate	EMC	623-53-0	3.70	3.80	yes	699	697	(662)	810	45	77	59			
Diethyl carbonate	DEC	105-58-8	4.58	4.64	yes	781	781	767	892	45	91	63	43	59	
Bis(2-methoxyethyl) ether	G2	111-96-9	6.18	6.28	yes	945	943	951	904	59	58	45	89	43	
Ethylene carbonate	EC	96-49-1	6.54	6.43	yes	960	960	(814)		88	43	44	58	42	
unknown			7.42	7.42	---	---	---	---	---	59	72	91	118		
Triethyl phosphate	TEP	78-40-0	7.78	7.89	yes	1125	1113	1132	883	99	155	127	109	82	81
Ethane-1,2-diyl diethyl dicarbonate	DEDD		9.33	9.42	yes	1321		(1303)	928	45	63	91	89	76	116
unknown			11.25	11.37	no	1587	---	---	---	89	117	45	44	70	63
unknown			13.59	13.72	no	1843	---	---	---	89	45	117	102	63	91
unknown			13.95	14.07	no	1870	---	---	---	89	117	45	147	70	87
unknown			17.50	17.63	no	---	---	---	---	89	117	45	70	102	44
unknown			19.53	19.64	no	---	---	---	---	89	117	45	59	72	102
**Electrolyte after storage**															
POF_3		13478-20-1		1.84	no				984	104	85	69	50		
Ethyl methyl carbonate	EMC	623-53-0	3.70	3.80	yes	699	697	(662)	810	45	77	59			
Diethyl carbonate	DEC	105-58-8	4.58	4.64	yes	781	781	767	892	45	91	63	43	59	
Ethylene carbonate	EC	96-49-1	6.54	6.43	yes	960	960	(814)		88	43	44	58	42	
unknown			7.42	7.42	no	---	---	---	---	59	72	91	118		
Triethylphosphat	TEP	78-40-0	7.78	7.89	yes	1125	1113	1132	883	99	155	127	109	82	81
Ethane-1,2-diyl diethyl dicarbonate	DEDD		9.33	9.42	yes	1321		(1303)	928	45	63	91	89	76	116
unknown			11.25	11.37	no	1587	---	---	---	89	117	45	44	70	63
unknown			13.59	13.72	no	1843	---	---	---	89	45	117	102	63	91
unknown			13.95	14.07	no	1870	---	---	---	89	117	45	147	70	87
unknown			17.50	17.63	no	---	---	---	---	89	117	45	70	102	44

unknown			19.53	19.64	no	---	---	---	---	89	117	45	59	72	102

[a] RT = Retention time
[b] Verified by measuring the pure substance exhibiting same retention time (±0.15 s) and same mass fragmentation
[c] RI values based on n-alkanes
[d] RI value determined earlier with pure compound (~ 5 µl/ml) or sample including the compound
[e] NIST based RI values for capillary columns (experimental) are provided for comparison. Values in brackets are estimated non-polar retention index values.
